# Does sex education before college protect students from sexual assault in college?

**DOI:** 10.1371/journal.pone.0205951

**Published:** 2018-11-14

**Authors:** John S. Santelli, Stephanie A. Grilo, Tse-Hwei Choo, Gloria Diaz, Kate Walsh, Melanie Wall, Jennifer S. Hirsch, Patrick A. Wilson, Louisa Gilbert, Shamus Khan, Claude A. Mellins

**Affiliations:** 1 Heilbrunn Department of Population & Family Health, Columbia University Mailman School of Public Health, New York, NY, United States of America; 2 Department of Sociomedical Sciences, Columbia University Mailman School of Public Health, New York, NY, United States of America; 3 Department of Psychiatry, Mental Health Data Science, Columbia University Medical Center and NY State Psychiatric Institute, New York, NY, United States of America; 4 Ferkauf Graduate School of Psychology, Yeshiva University, New York, NY, United States of America; 5 Department of Epidemiology, Columbia University Mailman School of Public Health, New York, NY, United States of America; 6 Social Intervention Group, Columbia University School of Social Work, New York, NY, United States of America; 7 Department of Sociology, Columbia University, New York, NY, United States of America; 8 Division of Gender, Sexuality and Health, Departments of Psychiatry and Sociomedical Sciences, New York State Psychiatric Institute and Columbia University, New York, NY, United States of America; University of Texas Medical Branch at Galveston, UNITED STATES

## Abstract

**Purpose:**

College-bound young people experience sexual assault, both before and after they enter college. This study examines historical risk factors (experiences and exposures that occurred prior to college) for penetrative sexual assault (PSA) victimization since entering college.

**Methods:**

A cross-sectional study, including an online population-based quantitiative survey with undergraduate students was conducted in spring 2016. Bivariate analyses and multivariable regressions examined risk and protective factors associated with ever experiencing PSA since entering college. Concurrently-collected in-depth ethnographic interviews with 151 students were reviewed for information related to factors identified in the survey.

**Results:**

In bivariate analyses, multiple historical factors were significantly associated with PSA in college including adverse childhood experiences and having experienced unwanted sexual contact before college (for women) and initiation of alcohol, marijuana, and sexual behaviors before age 18. Significant independent risk factors for college PSA included female gender, experiencing unwanted sexual contact before college, first oral sex before age 18, and “hooking up” (e.g., causual sex or sex outside a committed partnership) in high school. Receipt of school-based sex education promoting refusal skills before age 18 was an independent protective factor; abstinence-only instruction was not. In the ethnographic interviews, students reported variable experiences with sex education before college; many reported it was awkward and poorly delivered.

**Conclusions:**

Multiple experiences and exposures prior to college influenced the risk of penetrative sexual assault in college. Pre-college comprehensive sexuality education, including skills-based training in refusing unwanted sex, may be an effective strategy for preventing sexual assault in college. Sexual assault prevention needs to begin earlier; successful prevention before college should complement prevention efforts once students enter college.

## Introduction

Penetrative sexual assault (PSA) includes rape (defined as unwanted oral, anal, or vaginal penetration obtained by force or drug or alcohol incapacitation) and unwanted penetration due to verbal coercion. Nearly one in five (~18%) women in the US report lifetime exposure to rape and ~13% of women report lifetime exposure to unwanted penetration due to verbal coercion.^1^ Penetrative sexual assault (PSA), which refers to unwanted penetration due to all three methods, is a prevalent and pressing public health issue and often concentrated at younger ages. Among females who experience rape in the United States (U.S.), 80% experienced their first rape before the age of 25 and 42% before age 18; among men who experienced rape, 28% were first raped before age 10.[1] Among women, estimates of forcible rape during college range between 0.5–8.4% while estimates of incapacitated rape vary between 1.8–14.2%.[2]

Understanding risk and protective factors for PSA can strengthen prevention by targeting resources to areas of highest need, defining psychosocial factors (such as attitudes, skills, and behaviors) that can be changed, and pointing to opportunities for structural or early intervention programs. College groups at highest risk for experiencing PSA include female and gender-nonconforming (GNC), as well as lesbian, gay, bisexual, transgender, and questioning (LGBTQ) students.[3] Limited information is available on the risk of PSA for international students at U.S. universities.[4] Behavioral risk factors for PSA victimization include sexual behavior (multiple partners, sexual risk taking); substance misuse, particularly alcohol; and poor intimate partner relationship dynamics.[3] Risk factors for perpetrating PSA include attitudes associated with hostility towards women, rape myth acceptance, hypermasculinity, traditional gender roles, and acceptance of violence.[3,5]

Experiences from childhood and adolescence (before college) may influence PSA risk in college. For example, exposure to adverse childhood experiences (ACEs) and childhood sexual and physical abuse has been associated with increased risk for PSA in adolescence and adulthood.[6,7] Ports et al.[7] found that each ACE variable was significantly associated with adult sexual victimization, with childhood sexual assault being the strongest predictor of adult sexual victimization. Adolescent (pre-college) sexual assault is also significantly associated with PSA in college.[5,8–10] In national samples, 50% of adolescent and college women who report sexual assault also report revictimization.[11] ACEs also may have an indirect influence on PSA through their association with risky sexual behavior[12] and alcohol and drug use in adolescence and adulthood[13], which have both been associated with increased risk for PSA. [14,15] Earlier adolescent involvement in health risk behaviors such as drinking alcohol may increase risk for college sexual assault victimization and perpetration.[3,5] Exposure to pornography among high school and college-age adolescents has been associated with sexual assault perpetration.[16] These risk behaviors may also represent the adverse impact of childhood and adolescent social experiences.

Unlike risk factors for PSA, protective factors such as social and family support are not as well examined. Connectedness to social institutions such as school and family has been associated with reductions in sexual risk behaviors among youth[17] and may protect young people from sexual assault, however, sexual assault research has not explored this connection in-depth. Connectedness to religious institutions has also been associated with adolescent health but has not been explored in relation to sexual assault.[18]

Finally, vulnerability to sexual assault could be mitigated by policy and programmatic approaches such as comprehensive sexuality education (CSE) in middle and high school, which may include the teaching of sexual refusal skills as well as a broad range of other topics, and social and emotional learning interventions that explore gender. While CSE in middle and high school can reduce adolescent behaviors that lead to HIV, STIs, and unplanned pregnancy[19], research has not been conducted to measure the impact of pre-college CSE on sexual assault in college.[20] Promising intervention approaches include targeted interventions involving self-defense skills and resistance training for college[21,22] and high school[23] students and bystander programs[24,25], intended to improve environmental support for young people at risk of assault.

This paper examines the association of pre-college experiences and exposures with the risk of ever experiencing PSA during the undergraduate college years. We hypothesized that PSA in college would be negatively associated with exposure to sexuality education and connectedness to religion and positively associated with pre-college exposure to adverse childhood experiences, sexual assault, and pornography, and earlier initiation of health risk behaviors. Data come from our Sexual Health Initiative to Foster Transformation (SHIFT), a mixed-methods research project that examined risk and protective factors affecting sexual health and sexual violence among undergraduates at Columbia University (CU) and Barnard College (BC) in New York City.

## Methods

### Overview of survey data collection

This study analyzed data from a survey that was conducted online between March and May 2016 at Columbia University as a part of the Sexual Health Initiative to Foster Transformation (SHIFT). The majority of survey questions had been validated previously with college-age students (see below). The parts of the survey related to this analysis is available in S1 Appendix.

### Participants

Potential survey participants were selected using administrative records for 9,616 CU/BC undergraduate students age 18–29 years. Separate random samples were used to select undergraduates from CU (2,000) and from BC (500). Of these 2,500 students, 1,671 (67%) consented to participate (80.5% were from CU and 19.5% were from BC). The demographic characteristics of the stratified sample (i.e. gender, age, race/ethnicity, international status, year in school, financial aid status) were similar to the full CU/BC population.[26]

### Procedures

Study approval was obtained from the Columbia University Medical Center IRB; participants signed an online consent form and the project obtained a federal Certificate of Confidentiality to legally protect the data from subpoena as well as a waiver from CU to waive the obligation to report individual sexual assaults. A unique link was provided to all particpiants for them to access the survey at a location of their choosing (84% of participants) or at our on-campus research office (16% of participants). Participants provided informed consent on an electronic form and received $40 compensation. Participants were also entered into a lottery to win an additional $200 gift card.

Various methods were used to improve the response rate,[26] including email messages to generate interest; reminder emails to students who had been selected to participate using messages crafted to resonate with diverse student motives for participation (e.g., interest in sexual assault, compensation, community spirit, and achieving higher response rates than surveys at peer institutions); flyers; holding “study breaks” in which students were given snacks and drinks; and tabling in public areas on campus. On average, the survey took between 35 and 40 minutes to complete.

### Dependent and independent variables used in survey

Penetrative Sexual Assault. For our primary outcome, we adapted the Sexual Experiences Survey (SES), a measure of sexual assault victimization which has good internal consistency and validity.[27] The SES employs behaviorally specific questions, which capture the prevalence of sexual assault more accurately than questions that simply ask whether a person was sexually assaulted. The scale includes questions on type of assault, including sexualized touching without penetration (oral, anal or vaginal, other), attempted but not completed penetrative assault, and finally completed penetrative assault. We adapted the SES by combining separate questions on oral, anal, and vaginal penetration into a single question. Participants are also asked for each type of assault the method(s) of perpetration, including two types of verbal coercion, physical force, threats of physical harm, incapacitation, and other. This paper focuses on completed penetrative sexual assault (defined as oral, anal, vaginal or other penetration) perpetrated by verbal coercion, physical force, threats of physical harm, or incapacitation.

Demographic factors included age, gender (categorized for these analyses into three self-identified groups: female, male, and gender nonconforming or GNC),[28] race (American Indian/Alaskan Native, Asian, Black, Native Hawaiian/ Pacific Islander, White, other), Hispanic or Latino ethnicity, and sexual orientation (asexual, pansexual, bisexual, queer, homosexual, heterosexual, and other). Students could select multiple racial categories and on average selected 1.1 categories. Other childhood, adolescent, and family social and demographic variables included an indicator of family income (receipt of a Pell grant, a marker for lower income), if the student was born in the US (yes/no), where they grew up (urban, suburban, rural, other), type of high school (public, magnet, private, parochial, boarding school), high school gender composition (co-ed, single sex), parent’s educational attainment, and how long parents had lived in the US (never, whole life, part of life).

Connectedness to religion was measured as religious affiliation (including no affiliation) and attendance at religious services in high school.

Adverse childhood experiences. Six yes/no questions about adverse childhood experiences (physical abuse by parents or adults in household, sexual assault by parents or adults in household, parental divorce or separation, alcohol or drug use in the household, mental health or suicide in the household, or death of a family member) were adapted from the Adverse Childhood Experiences (ACE) study;[29] the ACE score reflected the number of ACEs reported. Students were also asked how often they viewed pornography in high school on a 6 point scale from never to every day or almost every day. Students were asked if they had ever experienced unwanted sexual contact before college.

Alochol and substance use. Participants were asked at what age they had first drunk more than a few sips of alcohol (later categorized as before age 15, age 15–17, age 18+ or never) and how often they drank in high school (never, few times per year, once a month, 2–3 times per month, once a week, twice a week, 3–4 times per week, 5–6 times per week, everyday). They were also asked age of first use for marijuana.

Sexual behavior. We asked students when they first engaged in oral, vaginal, and anal sex (later categorized as before age 15, age 15–17, and age 18+ or never) and about their relationships in high school (steady or serious relationships, one time hook-ups, ongoing hook-ups or friends with benefits). “Hooking up” and “friends with benefits” is often used ambiguously but includes causal sex or sex outside of a committed partnership. Oral and vaginal sex are often initiated in the same time period and generally before anal sex.[30] We preferentially examined age at first oral sex, to capture behaviors across sexual orientation groups.

Participants were asked 4 questions about sex education, i.e., formal education they had received at school before age 18 on how to say no to sex (refusal skills), methods of birth control, sexually transmitted diseases, and how to prevent HIV/AIDS. Abstinence-only instruction was defined as receiving refusal skills instruction but not receiving instruction about methods of birth control.

### Survey data analysis

Data were initially stratified into 3 strata: men, women, and GNC students; data on GNC students (n = 26) were not analyzed as the numbers were too low for statistical analysis and to avoid potential deductive identification.[31] Frequencies of all variables were produced for the whole sample and for women and men (Table 1).

**Table 1:** Frequency distribution of risk factors, SHIFT survey 2016.

		All		Men		Women	
		N	%	N	%	N	%
**Age**	18–19 years	1661	34.3	678	32.4	954	35.5
	20–22 years		55.5		57.2		57.2
	23–29 years		10.3		14.5		7.2
**Race**	American Indian or Alaska Native	1618	2.5	659	2.7	932	2.4
	Asian		28.5		27.0		29.6
	Black		13.5		11.4		14.6
	Native Hawaiian or Pacific Islander		1.4		1.4		1.3
	White or Caucasian		59.8		63.0		58.0
	Other		5.9		6.2		5.7
**Ethnicity**	Hispanic or Latino origin	1647	14.9	671	15.8	947	14.4
**Sexual orientation**	Asexual	1610	1.7	645	0.9	936	2.0
	Pansexual		1.7		0.9		2.0
	Bisexual		8.5		4.0		11.2
	Queer		4.2		1.4		4.5
	Heterosexual		80.3		85.3		78.7
	Homosexual		5.2		8.8		2.2
	Other		1.2		nr		1.7
**Childhood and Family**							
**Adverse Childhood Experiences**	None	1590	53.9	635	61.4	925	49.4
	1		23.8		21.3		25.7
	2		12.5		10.4		13.5
	3		6.4		3.9		7.9
	4		2.5		2.7		2.1
	5 or 6		0.9		nr		1.4
**Experienced unwanted sexual contact before college**	Ever experienced	1589	19.6	639	9.4	920	25.8
**Financial aid**	Pell grant	1587	22.8	639	22.2	921	22.9
**Student born in the United States**	Yes	1645	76.1	667	77.7	948	74.8
**Mothers Education**	No High School Diploma	1545	3.4	618	3.2	900	3.6
	High School Diploma		12.7		14.1		11.7
	Associate Degree		5.2		6.1		4.4
	Bachelor Degree		34.4		35.9		33.6
	Graduate Degree		44.3		40.6		46.8
**Mother lived in the US**	Whole life	1533	47.3	609	49.3	897	45.9
	Part of life lived in the US		42.6		38.6		45.3
	Never lived in US		10.1		12.2		8.8
**Any religious participation in high school**	Yes	1591	56.1	637	56.4	925	56.5
**Amount of Religious Participation in high school**	Daily	893	17.9	359	19.2	523	17.4
	Weekly		38.2		35.7		40.0
	Monthly		11.3		13.9		9.2
	Only on special occasions		32.6		31.2		33.5
**Alcohol and Other Drug Use**							
**Ever drank alcohol more than a few sips**	Before age 15	1618	14.9	647	17.2	940	13.5
	Age 15–17		39.9		41.9		38.4
	Age 18+ or never		45.2		41.0		48.1
**Alcohol use in high school**	I never drank any alcohol in High school	1588	44.3	633	41.9	926	46.0
	A few times per year		26.3		23.4		28.4
	Once a month		10.0		11.5		9.0
	2 to 3 times a month		10.5		11.8		9.6
	Once a week		4.9		6.8		3.7
	More than once a week		3.9		4.6		3.3
**Ever used marijuana**	Before age 15	1581	4.5	623	5.9	930	3.2
	Age 15–17		22.3		22.2		22.5
	Age 18+ or never		73.2		71.9		74.3
**Sexual Behavior**							
**Ever had oral sex**	Before age 15	1520	5.5	615	7.8	876	3.4
	Age 15–17		36.1		40.5		33.2
	Age 18+ or never		58.4		51.7		63.4
**Ever had vaginal sex**	Before age 15	1526	1.8	615	2.1	882	1.6
	Age 15–17		28.1		31.5		25.7
	Age 18+ or never		70.2		66.3		72.7
**Ever had anal sex**	Before age 15	1524	0.5	614	1.1	881	0.1
	Age 15–17		5.5		8.0		3.6
	Age 18+ or never		94.0		90.9		96.3
**Sexual experiences in high school**	Steady or serious relationship	1570	45.6	624	50.2	916	42.5
	Hook-up, one time		35.4		42.5		30.3
	Ongoing hook-up or friends with benefits		25.4		29.5		22.4
**Sex Education and Media Influences**							
**Formal instructions at school before age 18**	How to say no to sex	1590	54.0	636	55.5	925	53.1
	Methods of birth control		76.5		79.2		74.5
	Sexually transmitted diseases		86.7		86.6		86.6
	How to prevent HIV/AIDS		81.0		82.2		80.0
	Abstinence only instruction		3.6		1.6		5.0
**Viewed pornography in high school**	Once a month or less	1549	13.7	611	6.4	909	18.4
	2 or 3 days a month		9.8		8.3		10.6
	1 or 2 days a week		14.1		23.4		7.7
	3 to 5 days a week		14.5		30.9		3.7
	Every day or almost everyday		10.6		23.9		1.2
	Never		37.3		7.0		58.4

Note: Variables tested and not significantly related to PSA in bivariate analyses were dropped from Tables 1 and 2. These included where the student grew up (urban, suburban, rural), high school type (public, private), gender makeup of school, religious affiliation, primary source of health information, and father’s education and residence in U.S. Data on fathers was similar to data for mothers but had more missing data. Students could select multiple categories for certain questions: race, sex education before age 18, and sexual experiences in HS. NR = not reported if < = 3 cases in a cell.

Bivariate relationships between categorical predictor variables and PSA experienced since entering college were described using frequency tables, and assessed using χ^2^ or Fisher’s Exact Tests, as appropriate. Wilcoxon tests were used to assess associations between ordinal predictors and PSA. Bivariate associations were described, again for all subjects and for each of the 3 strata (Table 2). Variables that showed little relationship to PSA in bivariate analyses were deleted from Tables 1 and 2 but listed in the notes to Table 2. Multivariate logistic regression models of the log odds of ever experiencing PSA since entering college were fitted, with backward variable selection based on Bayesian Information Criteria (BIC), for all subjects, and for male and female students separately (Table 3). Finally, given intercorrelations among our independent variables we also examined associations among related predictor variables (e.g., ACE and ages at first oral, vaginal and anal sex). All analyses were performed using SAS version 9.4. Because of missing data, the analysis sample for specific variables was reduced.

**Table 2:** Percentage of students who had ever experienced penetrative sexual assault in college, SHIFT survey, 2016.

			All		Men		Women			
			%	p	%	p	%		p	
Overall Rates			10.1		5.2		13.6			
**Age**	18		7.3		3.9		9.8			
	22		12.7		6.5		16.7			
	23–29 years		5.1	0.1727	3.4	0.6658	7.7		0.0776	
**Race**	American Indian or Alaska Native		15.0	0.2889	11.8	0.2274	18.2		0.5257	
	Asian		5.3	< .0001	2.4	0.0529	7.2		0.0003	
	Black or African American		13.0	0.1385	4.1	0.7859	18.8		0.0587	
	Native Hawaiian or Pacific Islander		4.8	0.7152	0.0	1.0000	8.3		1.0000	
	White or Caucasian		11.8	0.0089	6.4	0.112	15.7		0.0300	
	Other		14.6	0.1415	4.9	1.0000	22.6		0.0480	
**Ethnicity**	Hispanic or Latino		12.6	0.1556	7.8	0.1635	16.5		0.2683	
**Sexual orientation**	Asexual		3.7	0.5135	16.7	0.2788	0.0		0.1548	
	Pansexual		29.6	0.0039	0.0	1.0000	36.8		0.0089	
	Bisexual		15.0	0.0520	11.5	0.1522	16.7		0.3357	
	Queer		20.9	0.0030	33.3	0.0091	23.8		0.0478	
	Heterosexual		8.7	0.0001	3.8	0.0003	12.4		0.0452	
	Homosexual		12.1	0.5597	14.3	0.0059	9.5		0.7563	
	Other		25.0	0.0448	66.7	0.0079	18.8		0.4682	
**Adverse Childhood Experiences**		None	7.8		4.1		11.0			
		1	9.2		5.9		11.3			
		2	14.7		10.6		16.8			
		3	21.0		8.0		26.4			
		4	12.5		0.0		21.1			
		5 or 6	26.7	< .0001	0.0	0.1183	30.8			0.0008
**Experienced unwanted sexual contact before college**		Ever experienced	19.9	< .0001	10.2	0.1127	22.8			< .0001
**Financial aid**		Pell grant	11.6	0.3402	8.0	0.0941	14.5			0.7338
**Student born in the United States**		Yes	11.4	0.0036	6.3	0.0216	15.0			0.0276
**Mothers Education**		No High School Diploma	17.3		10.0		21.9			
		High School Diploma	7.7		1.2		12.6			
		Associate Degree	3.7		2.6		5.0			
		Bachelor Degree	8.9		6.4		11.0			
		Graduate Degree	12.0	0.0248	6.0	0.2770	15.6			0.1054
**Mother lived in the US**		Whole life	13.1		6.4		17.8			
		Part of life lived in the US	8.6		5.6		10.6			
		Never lived in US	2.6	0.0001	1.4	0.2288	4.0			0.0005
**Any religious participation in high school**		Yes	9.7	0.5585	5.4	0.8943	12.9			0.5157
**Amount of Religious Participation in high school**		Daily	7.6		4.5		9.9			
		Weekly	8.9		5.6		11.0			
		Monthly	8.9		4.0		14.6			
		Only on special occasions	12.2	0.0900	6.3	0.6767	16.4			0.0753
**Ever drank alcohol more than a few sips**		Before age 15	14.8		10.0		19.4			
		Age 15–17	12.9		5.4		18.3			
		Age 18+ or never	6.4	< .0001	3.2	0.0273	8.4			< .0001
**Alcohol use in high school**		I never drank any alcohol in High school	5.9		3.1		7.9			
		A few times per year	12.7		6.8		16.4			
		Once a month	13.8		8.2		18.1			
		2 to 3 times a month	15.1		5.4		22.5			
		Once a week	12.8		4.7		23.5			
		More than once a week	13.6	< .0001	10.7	0.0654	17.2			< .0001
**Ever used marijuana**		Before age 15	28.6		18.9		41.4			
		Age 15–17	16.1		6.6		22.1			
		Age 18+ or never	7.2	< .0001	3.7	0.0003	9.7			< .0001
**Ever had oral sex**		Before age 15	16.9		8.3		31.0			
		Age 15–17	16.8		8.3		24.0			
		Age 18+ or never	6.2	< .0001	2.9	0.0131	8.2			< .0001
**Ever had vaginal sex**		Before age 15	18.5		0.0		35.7			
		Age 15–17	16.0		7.5		22.8			
		Age 18+ or never	8.2	< .0001	4.7	0.2749	10.6			< .0001
**Ever had anal sex**		Before age 15	0.0		0.0		0.0			
		Age 15–17	21.5		15.6		32.3			
		Age 18+ or never	10.1	0.0038	4.7	0.0075	13.7			0.0142
**Sexual experiences in high school**		Steady or serious relationship	12.4	0.0038	6.4	0.2246	17.2			0.0032
		Hook-up, one time	16.3	< .0001	9.4	< .0001	22.8			< .0001
		Ongoing hook-up or friends with benefits	16.4	< .0001	7.7	0.0934	23.9			< .0001
**Formal instructions at school before age 18**		How to say no to sex	8.0	0.0032	5.7	0.5542	9.6			0.0004
		Methods of birth control	10.0	0.9805	5.2	0.9291	13.6			0.7470
		Sexually transmitted diseases	9.9	0.7685	5.1	0.7899	13.3			0.8784
		How to prevent HIV/AIDS	9.7	0.3702	4.8	0.2895	13.2			0.7146
		Abstinence only instruction	5.3	0.2242	10.0	0.4179	4.4			0.0648
**Viewed pornography in high school**		Never	10.1		2.6		10.7			
		Once a month or less	14.2		2.6		16.2			
		2 or 3 days a month	10.5		2.0		15.6			
		1 or 2 days a week	6.9		2.1		17.4			
		3 to 5 days a week	9.9		7.5		24.2			
		Every day or almost everyday	11.2	0.2777	9.1	0.0575	45.5			0.0024

Notes: Percentages represent percentage in each strata having ever experienced penetrative sexual assault.

Variables tested and not significantly related to PSA in bivariate analyses were dropped from Tables 1 and 2. These included where the student grew up (urban, suburban, rural), high school type (public, private), gender makeup of school, religious affiliation, primary source of health information, and father’s education and residence in U.S.

Data on fathers was similar to data for mothers but had more missing data. Students could select multiple categories for certain questions: race, sex education before age 18, and sexual experiences in HS. NR = not reported if < = 3 cases in a cell.

**Table 3:** Multivariate model predicting penetrative sexual assaults, SHIFT survey, 2016.

Predictor	DF	ChiSq	p-value	Categories	OR	LCL	UCL
**All Subjects**								
Gender	2	26.0353	< .0001	Male	1.00	1.00	1.00
				Female	2.99	1.95	4.59
				Male	1.00	1.00	1.00
				Other gender	1.12	0.24	5.20
Age at first Oral Sex	2	18.0418	0.0001	Age 18+ or never	1.00	1.00	1.00
				Age 15–17	2.39	1.59	3.59
				Before age 15	2.38	1.15	4.94
Experienced unwanted sexual contact before college	1	9.4532	0.0021	No	1.00	1.00	1.00
				Yes	1.84	1.25	2.71
Experienced hook ups in high school	1	11.752	0.0006	No	1.00	1.00	1.00
				Yes	2.00	1.35	2.98
Received formal instructions in HS on how to say no to sex	1	9.43	0.0021	No	1.00	1.00	1.00
				Yes	0.57	0.40	0.82
**Men**								
Homosexual	1	9.2931	0.0023	No	1.00	1.00	1.00
				Yes	3.93	1.63	9.49
Sexual Orientation not listed	1	8.2317	0.0041	No	1.00	1.00	1.00
				Yes	44.35	3.33	591.54
Experienced hook ups in HS	1	12.5829	0.0004	No	1.00	1.00	1.00
				Yes	4.51	1.96	10.36
**Women**								
Age at First Oral Sex	2	29.4309	< .0001	Age 18+ or never	1.00	1.00	1.00
				Age 15–17	3.11	2.03	4.77
				Before Age 15	3.79	1.55	9.26
Prior to enrolling at Columbia/Barnard, did you experience unwanted sexual contact?	1	10.8233	0.001	No	1.00	1.00	1.00
				Yes	2.04	1.33	3.12
Received formal instructions in HS on how to say no to sex	1	11.6533	0.0006	No	1.00	1.00	1.00
				Yes	0.49	0.32	0.74

### Ethnographic data collection

The SHIFT team also included a 16-month ethnographic research component that included three methods of data collection: participant observation, focus groups, and in-depth interviews. As this paper focuses on historical risk factors we rely exclusively on the in-depth interivews, which gathered qualitative data on previous experiences (for an overview of the ethnographic methods see Hirsch et al. 2018[32]; Khan et al. 2018[33]. The research team conducted 151 in-depth ethnographic interviews among undergraduates about experiences of sexual assault. Follow up interviews were conducted with 26 of these study participants. Demographic information was collected on all subjects in order to purposively construct a sample that reflected the range of backgrounds present in the overall student body. Interviews were generally between one and a half and three hours in length. All interviews were done in a private office on campus; interviews were recorded, transcribed, and de-identified before being analyzed. Senior ethnographers trained interviewers as part of the pilot of the research instrument and continued to provide weekly feedback on the interview process. The ethnographic research team included seven members, representing diverse class and racial backgrounds, religions, and sexualities. Interview subjects could select their interviewer; subjects generally selected the person who initially recruited them. The senior ethnographers met weekly to discuss emerging findings and direct the team; the entire ethnographic team met at least weekly for two hours to discuss ethnographic research findings and to ensure continued and consistent training of interviewers. One of the senior ethnographers also met with the quantitative research team to integrate findings and coordinate activities. Subjects were recruited during participant observation or volunteered to be interviewed after receiving SHIFT fliers or other communications about the project. Ethnographic guides queried topics related to college experiences such as social relationships, adjustment to college, extracurricular activities, consensual sexual and drug use experiences, and sexual assault. The ethnographic interviews began with a series of questions about pre-college experiences, including family relationships; high school social, sexual, and drug experiences; and sources of information about sex including parents and sexuality education. Ethnographic findings in this paper are used to illustrate findings from the survey data and thus are confined to pre-college experiences. The ethnographic field guide is available in S2 Appendix.

## Results

### Characteristics of survey sample

Table 1 describes the frequency distribution of our sample including 678 men, 954 women, and 26 GNC students. Most were between the ages of 18–22 years; 15% were Latino or Hispanic; and 80% were heterosexual. Students identified with 1.1 racial categories on average.

Table 1 also describes childhood experiences and family background for CU/BC students. Slightly under half (46%) of undergraduates had reported 1 or more adverse childhood experiences and 23% reported 2 or more ACEs. ACEs were more common among women and GNC students than among men. Having experienced unwanted sexual contact before college was more common among women (26%) and GNC students (50%) than among men (9%).

Many CU/BC undergraduates were international students. Almost a quarter of students (24%) were born outside the U.S. and slightly more than half of mothers had lived outside the country; 10% of mothers never lived in the U.S. In general, mothers were well-educated. Education and residency data from mothers and fathers were similar, but data for fathers were more often missing, so only data for mothers is reported in Table 1. Religious participation in high school was reported by 56% of students; among those, 18% reported daily participation and a third reported participating only on special occasions.

Substance use and sexual activity were relatively varied in our sample. A sizeable number (44%) reported never drinking alcohol in high school. Only 15% had first tried alcohol before age 15 years and only 9% reported weekly high school drinking. Only 27% of participants reported marijuana use before age 18 and only 5% before age 15. Fewer than half of students initiated sexual behaviors before age 18, including oral sex (42%), vaginal sex (30%), and anal sex (6%). Undergraduates described high school romantic or sexual relationship experiences, including steady or serious relationships (46%), one-time hook-ups (35%), and on-going hook-ups (25%).

Students reported a wide range of exposure to formal instruction about sexuality, with over 50% having received formal education on sexual refusal (“how to say no to sex,” 54%), methods of birth control (77%), STIs (87%), and HIV/AIDS prevention (81%). Only 3.6% described receiving abstinence-only instruction as indicated by answering “Yes” on how to say no to sex and “No” on instruction about contraception. The frequency of viewing pornography in high school was highly varied, and more common among male and GNC students than women.

### Bivariate risk factors

Table 2 shows percentage of students experiencing PSA in college by pre-college characteristics. Overall, 10% of students reported experiencing PSA since starting college, including 5% of men, 14% of women, and 8% of GNC students. Groups reporting higher rates of PSA included white students (12%, compared to non-white students) and pansexual (30%) and queer (21%) students, compared to those who did not identify as pansexual or queer, respectively. Asian (5%) and heterosexual (9%) students were at lower risk, compared to non-Asians and non-heterosexual students, respectively.

PSA since entering college was associated with the number of ACES and unwanted sexual contact before college for all participants and for women specifically. Among all undergraduates and among men and women, PSA was higher among students who were born in the U.S.. Protective family factors included having a mother who had never lived in the U.S., or had lived only part of her life in the U.S., compared to living her whole life in the U.S (Never vs Whole Life: p = .0002; Part of Life vs Whole Life: p = .0086). The rate of PSA among those who never participated in religious services (11%) was similar to the rate among those who reported ever participating (10%).

Earlier initiation of sexual and substance use behaviors (before age 18 or before age 15, respectively) was associated with an increased risk of PSA in college, compared to those who initiated at 18+ years or never initiated. PSA was less likely among students who reported never drinking in high school (6%). High school relationship experiences were associated with increased risk of PSA including having a steady or serious relationship, a one-time hook up, or ongoing hook up or friends with benefits, when compared to students without each of these sexual experiences. Greater frequency of viewing pornography was associated with an increased risk of PSA in college for women and showed a borderline statistical association for men p = 0.0575).

Finally, formal instruction in high school about refusing sex (how to say no to sex) was significantly associated with reduced risk for PSA overall (8%) and among women (10%). However, having received most other features of school-based sex education before college was not associated with higher or lower risk of PSA in college; these included instruction about methods of birth control, sexually transmitted diseases, and how to prevention HIV/AIDS. Nor was abstinence-only instruction associated with risk of PSA.

### Multivariate risk factors

We next conducted multivariate analyses to examine independent risk factors for PSA (Table 3). In the overall group, risk factors included female gender identity (OR = 2.99), unwanted sexual contact before college (OR = 1.84), first oral sex before age 15 (OR = 2.38, compared to 18 or older), first oral sex between ages 15–17 (OR = 2.39, compared to 18 or older), and engaging in hooking up relationships in high school (OR = 2.00). Protective factors included receiving formal instruction in high school on refusing sex (how to say no to sex) (OR = 0.57). Independent risk factors among men for experiencing PSA in college included being homosexual (OR = 3.93) and hooking up in high school (OR = 4.51). For women, independent risk factors were earlier age at first oral sex (before age 15 [OR = 3.79]; between ages 15–17 [OR = 3.11], compared to 18 or older), and experience of unwanted sexual contact before college (OR = 2.04); a protective factor was receipt of formal education in high school on how to say no to sex (OR = 0.49).

### Supplemental analyses

Because of the possibility that certain independent variables were theoretically or statistically associated, we examined these associations to help us to understand and interpret our multivariable models (see S1 Table). We found that the timing of initiation of oral sex was highly associated with timing of other sexual and drug use behaviors; earlier initiation was also associated with more frequent viewing of pornography in high school. Hooking up in high school was highly correlated with watching porn and earlier initiation of oral sex. Also, sexual assault prior to college was highly correlated with other adverse childhood experiences. Similarly, some protective factors co-occurred; formal education about saying no to sex was highly correlated with receiving formal education about STIs, HIV/AIDS and contraception. However, formal education about saying no to sex was not associated with religious participation or religious affiliation suggesting that this refusal skills instruction was distinct from abstinence-only instruction.

### Ethnographic findings

Ethnographic interviews illustrate how family influences and sexual education were experienced by young people and suggest some of the dynamics of the relationship between substance use and sexual activity. Many students reported limited or no discussions of sex or sexuality with family, as well as fear of the consequences of initiating such discussions. As one young man from a conservative religious background told us,

*“My dad went to a Presbyterian college … I went to a very small Christian evangelical high school*, *where if I had come out, I probably would've been kicked out … so that shaped, definitely, how I express myself …”*

A woman from a similar background noted that she, “*never had a whole birds and bees talk from them*. *We just didn't ever talk about it* … *So*, *still to this day*, *I don't think I've had [laughs] sex ed*.*”*

Our quantitative findings may over or under-estimate the impact of sexual education (or lack of such education), because, as illustrated by this ethnographic research, the content of that education is so mixed. A woman who went to public school described sex education as focused on abstinence and mixed with incomplete information about STIs and condom use

*“So in our health class we had some sex ed*. *It was poorly delivered. We're supposed to have like your talk about birth control options and that never happened. we had an abstinence educator come in … and tell us all that if we have sex, we'll be like soiled forever and no one will love us.”*

Those students who experienced more comprehensive programs generally described sex ed as awkward and focused on STIs, HIV, and pregnancy and contraception (mostly condoms). Even among students who received more comprehensive education, few described receiving education about sexual assault. A Black woman from a wealthy family noted,

*“It was pretty comprehensive … once or twice a year*, *we would have a nurse or somebody who would come and talk to us about sex and puberty. But, it was very much from a medical perspective, “This is what your period is; this is what birth control is. This is what reproduction looks like.” But we didn’t learn about sexual violence or things like that.”*

The lack of institutional education led students to have ad hoc solutions to learn more about sex and sexuality. Commonly students relied upon older siblings or pornography, and even the students themselves acknowledged that were imperfect sources of information. As one young man told us, “*We had friends who talked about it and joked about it*, *but none of us had really any idea what it was*.*”*

Regardless of when students had sexual intercourse for the first time (for most this was during senior year of high school, the summer between high school and college or their first semester at college), they commonly spoke of wanting to “get it over with”. Many students who had initiated sex before being interviewed specifically expressed not wanting to be a virgin in college. As one young man told us, *“So I mean like I was a virgin coming to school and like I wanted that to change [laughs]* …*”* Other students lost their virginity in long-term relationships. One young woman reported, *“So my first boyfriend—it was like we were both virgins—that was like a comforting thing*. *I trusted him a lot*. *Totally*.*”*

In addition to wanting sexual experiences before college, students often wanted to increase their experience with drinking and substance use. Just as with sex, students did not have much explicit guidance from school or family, and so they took it upon themselves to develop experiences themselves. *“I didn’t want to come in and just look like an idiot in front of my teammates because*, *I’ve never drank*,*”* a female athlete told us.

The experiences of sex and substance were paired for some students, most frequently leading to students finding ways to hide their activities rather than talk with them with parents or other mentors. As one young man described his high school sexual encounters,

*“So*, *we always would smoke first* [marijuana before sex] *which meant we would have to go somewhere where that was okay too*. *So there's this parking garage at a marina that we would go to that like underground*, *dark*, *and I had a little Camaro so I could get in between two cars; nobody can see you*.*”*

In sum, many students reported that sex education before college was awkward and poorly delivered. Even among students who did receive more comprehensive education in high school, few described sexual education addressing sexual assault.

## Discussion

We found that multiple social and personal factors prior to college were associated with experiencing sexual assault in college, including gender, experience of ACEs and unwanted sexual contact before college, hooking up in high school, initiation of oral sex before age 18, and sexual orientation (for men). Our analyses also showed that having received formal education about how to say no to sex (refusal skills training) before age 18 was a protective factor against penetrative sexual assault in college; it is important to note that the vast majority of those who received instruction in refusal skills also received other forms of sexual education. Other aspects of sexuality education and receipt of abstinence-only instruction were not significantly related to sexual assault. Our ethnographic data underline the diversity in exposure to sex education before college and the fact that the quality of sex education was mixed, and often poor.

Our study reaffirmed existing research about pre-college sexual assault as a risk factor for sexual assault in college.[5,11] Although the prevalence of revictimization has been well documented, the mechanism is unclear and perhaps represents a nexus of adverse experiences. ACEs–including verbal, physical, and childhood sexual abuse–may increase the risk of sexual assault in adulthood and early initiation of drug and alcohol use, which may also increase the likelihood of PSA in adulthood.[7]Although experiencing even a single sexual assault is associated with negative outcomes such as substance use, interpersonal issues and psychological issues,[11] revictimization poses an even greater risk, especially for post-traumatic stress disorder (PTSD)[11] and depression.[34] The increased risk for those who have been victimized underlines the importance of screening high school and college students in settings such as primary care settings where many of them are likely to go at least annually[35] and ensuring that comprehensive mental health services are available for those with a history of sexual assault.

We found initiation of sex and alcohol use before age 15 or age 18 was associated with PSA in college. Earlier initiation of sexual intercourse (before age 15) has been associated with childhood adverse experiences[36] and with sexual risk taking later in life.[37] Thus, earlier initiation of sex or alcohol may be a consequence of childhood adversity and related to later risk taking propensity. Earlier initiation of either behavior may be part of a sequence of events leading to sexual assault or perhaps a marker for an underlying trait such as risk-taking. Our ethnographic data suggests students often initiated intercourse (and alcohol use) as a way to prepare for college life.

Certain childhood and family factors were found to be protective against experiencing sexual assault in college. Being foreign born (as a student) and having parents who never lived in the U.S. (indicators of being an international student) were protective against PSA. Religious affiliation showed no association with PSA, however, participation in religious services in high school showed nuanced findings. Among those who participated at all in religious services, greater frequency of religious participation in high school was protective. However, when simply comparing the rate of PSA among those who never participated in religious services to that of those who did participate, the rate was nearly identical. This may be indicative of selection effects, or of the protective impact of more substantial participation in a faith community. Ethnographic interviews underline the complexity of religious impacts on vulnerability; the student who referred to his Christian evangelical high school to denote a milieu in which it was not possible to come out to his parents (thus depriving him of potential parental social support) suggest that religion may also increase risk by preventing young people from openly communicating about sex. Being foreign born or having immigrant parents may reflect connectedness to family leading to less engagement in health risk behaviors. However, further work is needed to understand both of these findings.

Research has shown the importance of comprehensive sexual health education in secondary school for reduction of sexual risk behaviors among adolescents;[19] published research on comprehensive sexuality education before college has not assessed the impact on sexual assault in college. Thus, the title of our paper asks: “Does sex education before college protect students from sexual assault in college?” Our data suggests that adolescents who receive refusal education before college (but not other aspects of sex education) are at decreased risk of PSA during college. This finding is consistent with recent research on sexual assault prevention, including feminist self-defense and resistance training among high school and college students.[21–23] A recent review suggests that K-12 comprehensive sex ed has the qualities of effective prevention programs and has the potential to mitigate the risk factors associated with sexual violence perpetration—by starting prevention early in the lifecourse.[38]

We found notable differences in the experience of sexual assault between college men and women; unfortunately, the number of GNC students was too low to allow analysis. Consistent with previous studies, women were much more likely to experience penetrative sexual assault (14%) compared to men (5%). Likewise, independent risk factors (in Table 3) for having experienced PSA were different for women and men.

Data from qualitative interviews were used to illustrate findings from our quantitative survey. Importantly, we did not find qualitative data that contradicted our quantitative survey findings. While the data across the two methods were consistent, the qualitative data pointed to dimensions that might be further evaluated in future research. In particular the qualitative data revealed that those students who reported receiving formal sexual education had enormously varied experiences. Many students received education that was primarily “fear-based.” Future research might explore the effects of different aspects of sexual education on health outcomes.

There are several limitations to these data and this analysis. The first is that the data for this study come from two connected schools in one city and therefore may not be generalizable to other colleges and universities or to youth who are not enrolled in college. Although the survey had a good response rate, some selection bias may have occurred, as well as the bias that is inherent in self-reported data. The ethnographic data come from a small sample with data that might not reflect the larger whole. That said, we did not find qualitative data that contradicted our quantitative survey. Our data are cross-sectional and therefore identifying causality is difficult. Likewise, recall bias may occur after the trauma of sexual assault. Our survey also had limited detail about sexuality education received such as the timing, number of sessions, and other aspects of health sexuality. Finally, the number of GNC student was too small to allow examination.

### Implications and contributions

This study has important implications for policy and further research. In the broadest sense, our findings point to the underexplored opportunities for pre-college sexual assault prevention, including work with families, K-12 educational institutions, and religious communities. Sexual assault before college enrollment has consistently been associated with sexual assault in college, including in our study, highlighting the need for prevention efforts. Effective prevention before students arrive at college may help prevent sexual assault in college. Support to families during adolescence to prevent ACE and to communicate about adolescent sexuality and drug use may also help prevent sexual assault.

More research is needed on how pre-college educational and skill-building efforts can augment efforts now taking place during college to prevent sexual assault. Importantly, it suggests that sex education promoting refusal skills before college may protect young people in college. Sexual assault prevention should adopt a lifecourse perspective, including teaching young people before college about healthy and unhealthy sexual relationships and how to say no when sexual interaction is not wanted and yes when it is wanted. Education in college—including bystander training and sexual refusal skills—should be provided, particularly those students who may not have received such education before entering college.

## Supporting information

S1 Table Cross tabulations of independent variables. (DOCX)

S1 Appendix Quantitative survey questions. (DOCX)

S2 Appendix SHIFT in-depth interview guide. (PDF)
