# Supplementary material for: Does sex education before college protect students from sexual assault in college?
Source: PLoS One. 2018 Nov 14;13(11):e0205951. doi: 10.1371/journal.pone.0205951 (PMC6235267; doi:10.1371/journal.pone.0205951)
Supplement: S1 Table — (DOCX) [file pone.0205951.s001.docx]

**Supplemental Table 4. Cross tabulations of Independent Variables, Female Undergraduates**

| **Age of initiation of oral sex** |  |  |  |  |  |  |
| --- | --- | --- | --- | --- | --- | --- |
|  | | **< Age 15** | **Age 15-17** | **Age 18+** | **Spearman** | **p-value** |
| **Variable** | **Level** | **%** | **%** | **%** | **rho** | **Prob** |
| Ever had vaginal sex before age 18 | Before Age 15 | 33.3 | 1.0 | 0.18 | . | . |
|  | Age 15-17 | 53.3 | 66.2 | 3.24 | . | . |
|  | Age 18 or Later | 13.3 | 32.8 | 96.58 | 0.72 | <.0001 |
| Ever had anal sex before age 18 | Before Age 15 | 3.3 | 0.0 | 0 | . | . |
|  | Age 15-17 | 23.3 | 8.7 | 0 | . | . |
|  | Age 18 or Later | 73.3 | 91.4 | 100 | 0.28 | <.0001 |
| Ever Aged marijuana before age 18 | Before Age 15 | 36.7 | 5.6 | 0.18 | . | . |
|  | Age 15-17 | 40.0 | 43.9 | 13.19 | . | . |
|  | Age 18 or Later | 23.3 | 50.5 | 86.63 | 0.44 | <.0001 |
| Ever drank alcohol more than a few sips before age 18 | Before Age 15 | 56.7 | 19.9 | 8.15 | . | . |
|  | Age 15-17 | 33.3 | 55.0 | 30.98 | . | . |
|  | Age 18 or Later | 10.0 | 25.1 | 60.87 | 0.38 | <.0001 |
| When you were in high school, how frequently did you view pornographic material? | Once a month or less | 23.3 | 21.3 | 17.16 | . | . |
|  | 2 or 3 days a month | 16.7 | 12.9 | 9.59 | . | . |
|  | 1 or 2 days a week | 13.3 | 8.4 | 7.56 | . | . |
|  | 3 to 5 days a week | 3.3 | 4.9 | 3.51 | . | . |
|  | Every day or almost everyday | 3.3 | 2.5 | 0.55 | . | . |
|  | Never | 40.0 | 50.0 | 61.62 | -0.13 | 0.0002 |

| **Experience of unwanted sexual contact before college** |  |  |  |  |
| --- | --- | --- | --- | --- |
|  | | **Yes** | **No** | **p-value** |
| **Variable** | **Level** | **%** | **%** | **Pr > \|t\|** |
| Count of Adverse Childhood Events | 0 | 27.4 | 56.9 | . |
|  | 1 | 29.1 | 24.6 | . |
|  | 2 | 19.7 | 11.5 | . |
|  | 3 | 15.8 | 5.0 | . |
|  | 4 | 4.7 | 1.2 | . |
|  | 5 | 3.0 | 0.7 | . |
|  | 6 | 0.4 | 0.0 | <.0001 |
| While you were growing up...Did a parent or other adult in the household [often hurt you]? | Yes | 21.4 | 8.8 | <.0001 |
| While you were growing up...Did an adult or person at least 5 years older [ever attempt or actually have sex with you]? | Yes | 27.4 | 2.8 | <.0001 |
| While you were growing up...Were your parents ever separated or divorced? | Yes | 37.0 | 21.5 | <.0001 |
| While you were growing up...Did you live with anyone who was a problem drinker or alcoholic or who abused drugs? | Yes | 22.2 | 9.8 | <.0001 |
| While you were growing up...Was a household member depressed or mentally ill, or did a household member attempt suicide? | Yes | 34.6 | 19.4 | <.0001 |
| While you were growing up...Did a household member pass away? | Yes | 10.7 | 9.2 | 0.4816 |

| **Experienced hook ups in high school** |  |  |  |  |
| --- | --- | --- | --- | --- |
|  | | **Yes** | **No** | **p-value** |
| **Variable Label** | **Level** | **%** | **%** | **Pr > \|t\|** |
| While in high school did you have a[...]...Steady or serious relationship | Yes | 58.6 | 35.3 | <.0001 |
| Ever had oral sex before age 18 | Before Age 15 | 8.5 | 1.0 | . |
|  | Age 15-17 | 60.7 | 20.6 | . |
|  | Age 18+ | 30.7 | 78.3 | <.0001 |
| When you were in high school, how frequently did you view pornographic material? | Once a month or less | 24.8 | 15.5 | . |
|  | 2 or 3 days a month | 14.2 | 8.9 | . |
|  | 1 or 2 days a week | 6.9 | 8.1 | . |
|  | 3 to 5 days a week | 4.4 | 3.6 | . |
|  | Every day or almost everyday | 1.1 | 1.3 | . |
|  | Never | 48.5 | 62.6 | 0.0012 |

| **Received formal instructions at school on saying no to sex** |  |  |  |  |
| --- | --- | --- | --- | --- |
|  | | **Yes** | **No** | **p-value** |
| **Variable** | **Level** | **%** | **%** | **Prob** |
| Before age 18, received formal instructions at school about...Methods of birth control | Yes | 90.6 | 56.2 | <.0001 |
| Before 18, received formal instructions at school about...Sexually transmitted diseases | Yes | 97.8 | 74.0 | <.0001 |
| Before 18, received formal instructions at school about...How to prevent HIV/AIDS | Yes | 95.7 | 62.2 | <.0001 |
| Participation in religious/spiritual services in high school | Daily | 8.5 | 11.6 | . |
|  | Weekly | 23.9 | 21.1 | . |
|  | Monthly | 5.8 | 4.6 | . |
|  | Only on special occasions | 19.2 | 18.5 | . |
|  | Never | 42.7 | 44.2 | 0.9832 |

***Supplemental Table 4. Cross tabulations of Independent Variables, Male Undergraduates***

| **Age of initiation of oral sex** |  |  |  |  |  |  |
| --- | --- | --- | --- | --- | --- | --- |
|  | | **<15** | **15-17** | **18+** | **Spearman** | **p-value** |
| **Variable** | **Level** | **%** | **%** | **%** | **rho** | **Prob** |
| Ever had vaginal sex before age 18 | Before Age 15 | 27.1 | 0.0 | 0.0 | . | . |
|  | Age 15-17 | 43.8 | 64.7 | 3.8 | . | . |
|  | Age 18 or Later | 29.2 | 35.3 | 96.2 | 0.65 | <.0001 |
| Ever had anal sex before age 18 | Before Age 15 | 14.6 | 0.0 | 0.0 | . | . |
|  | Age 15-17 | 20.8 | 15.7 | 0.0 | . | . |
|  | Age 18 or Later | 64.6 | 84.3 | 100.0 | 0.36 | <.0001 |
| Ever Aged marijuana before age 18 | Before Age 15 | 20.0 | 9.2 | 2.0 | . | . |
|  | Age 15-17 | 26.7 | 33.1 | 15.1 | . | . |
|  | Age 18 or Later | 53.3 | 57.7 | 82.9 | 0.29 | <.0001 |
| Ever drank alcohol more than a few sips before age 18 | Before Age 15 | 37.5 | 21.5 | 11.8 | . | . |
|  | Age 15-17 | 35.4 | 54.3 | 34.5 | . | . |
|  | Age 18 or Later | 27.1 | 24.3 | 53.7 | 0.29 | <.0001 |
| When you were in high school, how frequently did you view pornographic material? | Once a month or less | 4.2 | 4.2 | 8.5 | . | . |
|  | 2 or 3 days a month | 6.3 | 8.4 | 8.2 | . | . |
|  | 1 or 2 days a week | 20.8 | 27.3 | 22.3 | . | . |
|  | 3 to 5 days a week | 41.7 | 29.4 | 31.5 | . | . |
|  | Every day or almost everyday | 27.1 | 26.9 | 21.0 | . | . |
|  | Never | 0.0 | 3.8 | 8.5 | -0.11 | 0.0056 |

| **Experience of unwanted sexual contact before college** |  |  |  |  |
| --- | --- | --- | --- | --- |
|  | | **Yes** | **No** | **p-value** |
| **Variable** | **Level** | **%** | **%** | **Pr > \|t\|** |
| Count of Adverse Childhood Events | 0 | 35.6 | 64.2 | . |
|  | 1 | 28.8 | 20.6 | . |
|  | 2 | 17.0 | 9.4 | . |
|  | 3 | 8.5 | 3.5 | . |
|  | 4 | 8.5 | 2.1 | . |
|  | 6 | 1.7 | 0.2 | <.0001 |
| While you were growing up...Did a parent or other adult in the household [often hurt you]? | Yes | 25.4 | 9.8 | 0.0003 |
| While you were growing up...Did an adult or person at least 5 years older [ever attempt or actually have sex with you]? | Yes | 17.2 | 2.3 | <.0001 |
| While you were growing up...Were your parents ever separated or divorced? | Yes | 33.9 | 15.6 | 0.0004 |
| While you were growing up...Did you live with anyone who was a problem drinker or alcoholic or who abused drugs? | Yes | 20.3 | 10.6 | 0.025 |
| While you were growing up...Was a household member depressed or mentally ill, or did a household member attempt suicide? | Yes | 20.3 | 13.2 | 0.1298 |
| While you were growing up...Did a household member pass away? | Yes | 15.3 | 8.2 | 0.0711 |

| **Experienced hook ups in high school** |  |  |  |  |
| --- | --- | --- | --- | --- |
|  | | **Yes** | **No** | **p-value** |
| **Variable** | **Level** | **%** | **%** | **Pr > \|t\|** |
| While in high school did you have a[...]...Steady or serious relationship | Yes | 62.8 | 40.6 | <.0001 |
| Ever had oral sex before age 18 | Before Age 15 | 15.1 | 2.7 | . |
|  | Age 15-17 | 57.1 | 26.8 | . |
|  | Age 18+ | 27.8 | 70.6 | <.0001 |
| When you were in high school, how frequently did you view pornographic material? | Once a month or less | 5.1 | 7.3 | . |
|  | 2 or 3 days a month | 9.4 | 7.9 | . |
|  | 1 or 2 days a week | 27.0 | 21.6 | . |
|  | 3 to 5 days a week | 30.9 | 30.9 | . |
|  | Every day or almost everyday | 24.6 | 22.7 | . |
|  | Never | 3.1 | 9.6 | 0.1613 |

| **Received formal instructions at school on saying no to sex** |  |  |  |  |
| --- | --- | --- | --- | --- |
|  | | **Yes** | **No** | **p-value** |
| **Variables** | **Level** | **%** | **%** | **Prob** |
| Before age 18, received formal instructions at school about...Methods of birth control | Yes | 97.2 | 56.9 | <.0001 |
| Before 18, received formal instructions at school about...Sexually transmitted diseases | Yes | 99.7 | 70.3 | <.0001 |
| Before 18, received formal instructions at school about...How to prevent HIV/AIDS | Yes | 98.0 | 62.5 | <.0001 |
| Participation in religious/spiritual services in high school | Daily | 9.5 | 12.1 | . |
|  | Weekly | 20.4 | 20.0 | . |
|  | Monthly | 7.5 | 8.6 | . |
|  | Only on special occasions | 17.5 | 17.5 | . |
|  | Never | 45.1 | 41.8 | 0.3239 |
