# Supplementary material for: Does sex education before college protect students from sexual assault in college?
Source: PLoS One. 2018 Nov 14;13(11):e0205951. doi: 10.1371/journal.pone.0205951 (PMC6235267; doi:10.1371/journal.pone.0205951)

**Appendix:
Quantitative Survey: Items Used in this Analysis**

**Predictor Variables:**

Q1 How old are you?

- 18 ____________________
- 19
- 20
- 21
- 22
- 23
- 24
- 25
- 26 or older
- Refuse to answer

Q2 What is your current gender identity?

- Male
- Female
- TransMale/Transman
- TransFemale/Transwoman
- Genderqueer or gender non-conforming
- Other (please specify) ____________________
- Refuse to answer

Q3 What sex were you assigned at birth?

- Male
- Female
- Intersex
- Refuse to answer

Q4 Are you of Hispanic or Latino origin?

- Yes
- No
- Refuse to answer

Q5 What is your race? (Select all that apply)

- American Indian or Alaska Native
- Asian
- Black or African American
- Native Hawaiian or Pacific Islander
- White or Caucasian
- Other (please specify) ____________________
- Refuse to answer

Q6 Were you born in the United States?

- Yes
- No
- Refuse to answer

Q9 Which of the following best describes the place where you grew up? If you lived in multiple places, please select the one where you spent the most time.

- Urban
- Suburban
- Rural
- Other (please specify) ____________________
- Refuse to answer

Q30 What is your religious faith or belief? (Select all that apply)

- Buddhism
- Christianity
- Islam
- Judaism
- Hinduism
- Sikhism
- Spiritual, but not religious
- Other (please specify) ____________________
- None
- Refuse to answer

Q31 How often do you participate in your religious/spiritual services or practices?

- Daily
- Weekly
- Monthly
- Only on special occasions
- Never
- Refuse to answer

Q32 Please check if you receive any of the following kinds of financial aid. (Select all that apply)

- Pell grant
- Federal
- State/Local
- Institutional
- Columbia/Barnard need based aid
- Other (please specify) ____________________
- None
- Refuse to answer

Q47 How old were you when you had your first drink of alcohol other than a few sips?

- I have never had a drink of alcohol or have only ever had a few sips
- Less than 12 years
- 13-14 years old
- 15-17 years old
- 18-20 years old
- 21 or older
- Refuse to answer

Q56 Please indicate whether you have ever used the following, and if so, whether you have used it since entering Columbia/Barnard, and/or in the last 30 days. (Select all that apply). You MUST choose one response for each column. If you haven't used any of the following in the designated time period, please select the response option "None of the above" for the appropriate column.


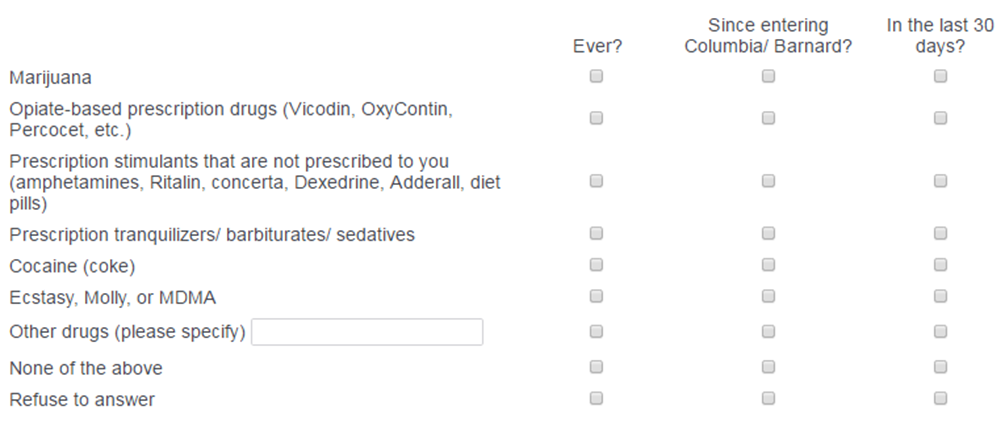


Q64 What is your sexual orientation?

- Asexual
- Pansexual
- Bisexual
- Queer
- Heterosexual
- Homosexual
- A sexual orientation not listed here (please specify) ____________________
- Refuse to answer


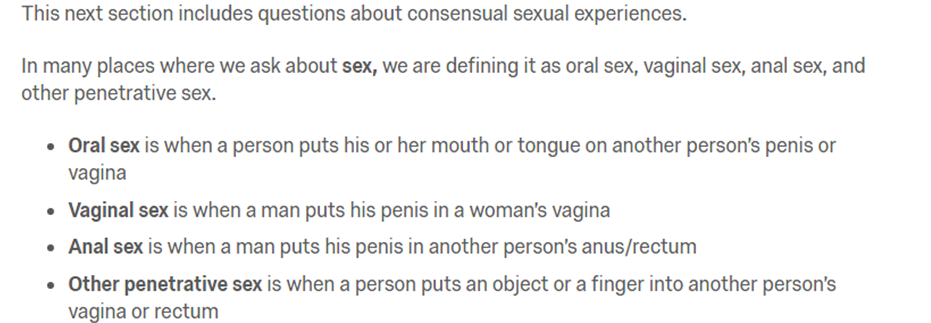


Q67 Which of these have you ever done? (Select all that apply.)

- Kissed or made out
- Touched someone's breast, chest, or buttocks area with your hand
- Had your breast or buttocks area touched by someone's hand
- Stimulated someone's genitals with your hand
- Had your genitals stimulated by someone's hand
- Performed oral sex on someone
- Had someone perform oral sex on you
- Had vaginal sexual intercourse
- Had anal intercourse: you penetrated someone
- Had anal intercourse: you were penetrated by someone
- Masturbated or stimulated your own genitals
- Had an orgasm
- None
- Refuse to answer

Q92 When you were in high school, how frequently did you view pornographic material (such as magazines, movies, and/or Internet sites)?

- Once a month or less
- 2 or 3 days a month
- 1 or 2 days a week
- 3 to 5 days a week
- Every day or almost everyday
- Never
- Refuse to answer

Q225 Prior to enrolling at Columbia/Barnard, did you experience unwanted sexual contact?

- No
- Yes
- Refuse to answer

Q228 The following questions ask about stressful or traumatic events that may have occurred in your childhood or adolescent years. Remember that your responses are completely confidential. At the end of the survey you’ll be provided with support resources if you’d like to speak with someone. While you were growing up:


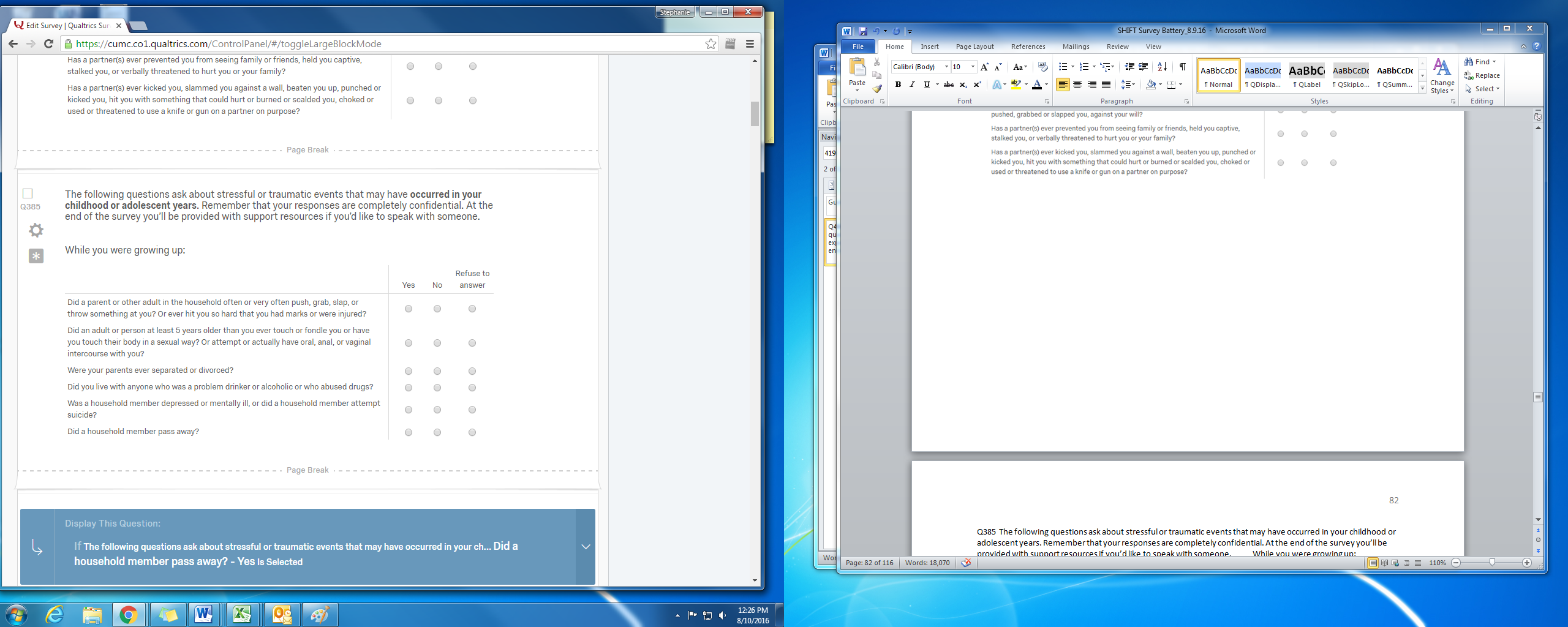


Q249 Which of the following best describes the high school from which you graduated.

- Public high school open to all people who lived where I did
- Public high school, competitive entry or magnet school
- Private high school, not boarding school
- Parochial school, not boarding school
- Boarding school
- Refuse to answer

Q250 What was the gender make-up of the high school you attended?

- Co-ed
- Single sex
- Refuse to answer

Q251 While in high school, how often did you participate in religious/spiritual services or practices?

- Daily
- Weekly
- Monthly
- Only on special occasions
- Never
- Refuse to answer

Q252 While in high school did you have any of the following sexual/ romantic relationships?


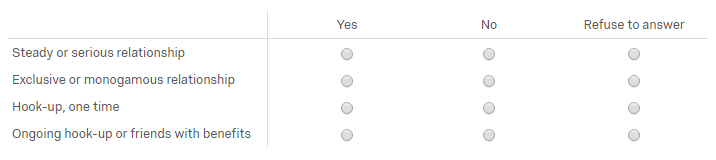


Q253 In general, when you were in high school, how often did you usually have any kind of drink containing alcohol?

- I never drank any alcohol in High school
- A few times per year
- Once a month
- 2 to 3 times a month
- Once a week
- Twice a week
- 3 to 4 times a week
- 5 to 6 times a week
- Every day
- Refuse to answer

Q254 Before you were 18, did you ever have any formal instructions at school about:


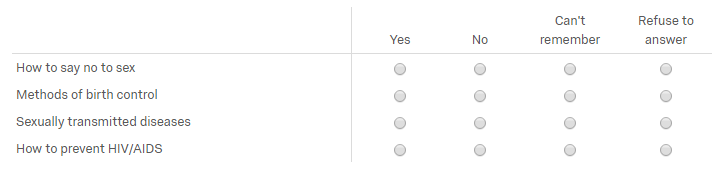


Q255 Where did most of your knowledge about contraception and safe sex come from? (Select all that apply)

- I have no knowledge of contraception or sexual health
- Parents/guardian
- Other family member (brother, sister, cousin, etc.)
- School
- Friend(s)
- Doctor, nurse, or other healthcare professional
- Media (e.g. magazines, Internet, TV, books)
- Other (please specify) ____________________
- Refuse to answer

Q257 What is your relationship with Caregiver #1?

- Birth parent
- Adoptive parent
- Foster parent
- Other relative
- Other (please specify) ____________________
- Refuse to answer

Q258 What is the gender of Caregiver #1?

- Male
- Female
- TransMale/Transman
- TransFemale/Transwoman
- Gerderqueer or gender non-conforming
- Other (please specify) ____________________
- Refuse to answer

Q259 What level of education has Caregiver #1 completed?

- No High School Diploma
- High School Diploma
- Associate Degree
- Bachelor Degree
- Graduate Degree
- Unknown
- Refuse to answer

Q260 Was Caregiver #1 born in the US?

- Yes
- No
- Refuse to answer

Q261 Please indicate the number of years Caregiver #1 has lived in the US.

- Whole life
- If not whole life, number of years (please write in): ____________________
- Never lived in US
- Refuse to answer

Q262 What is your relationship with Caregiver #2?

- Birth parent
- Adoptive parent
- Foster parent
- Other relative
- Other (please specify) ____________________
- I do not have a second caregiver
- Refuse to answer

Q263 What is the gender of Caregiver #2?

- Male
- Female
- TransMale/Transman
- TransFemale/Transwoman
- Gerderqueer or gender non-conforming
- Other (please specify) ____________________
- Refuse to answer

Q264 What level of education has Caregiver #2 completed?

- No High School Diploma
- High School Diploma
- Associate Degree
- Bachelor Degree
- Graduate Degree
- Unknown
- Refuse to answer

Q265 Was Caregiver #2 born in the US?

- Yes
- No
- Refuse to answer

Q266 Please indicate the number of years Caregiver #2 has lived in the US

- Whole life
- If not whole life, number of years: ____________________
- Never lived in US
- Refuse to answer

**Outcome Variables for this paper:**

The following questions concern sexual contact that you may have had that you did not want. Unwanted sexual contact is sexual contact that you did not consent to or agree to and that you did not want to happen. We know these are personal questions, so please remember your information is completely confidential and will not be connected to your name.  We hope this helps you to feel comfortable answering each question. We want to know about your experiences since you enrolled at Columbia/Barnard. These experiences could have occurred on or off campus, when school was in session or when you were on a break. If several experiences occurred on the same occasion—for example, if one night someone told you some lies and had sex with you when you were too drunk, you should indicate both.

161 Someone had oral, anal, vaginal, or other penetrative sex me without my consent or agreement by (Select all that apply):


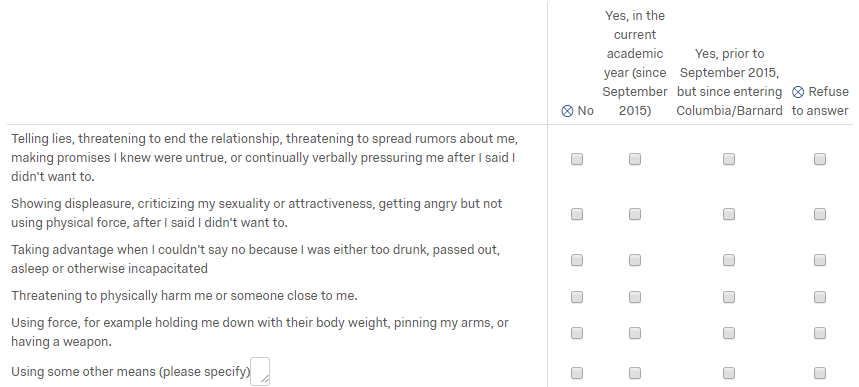


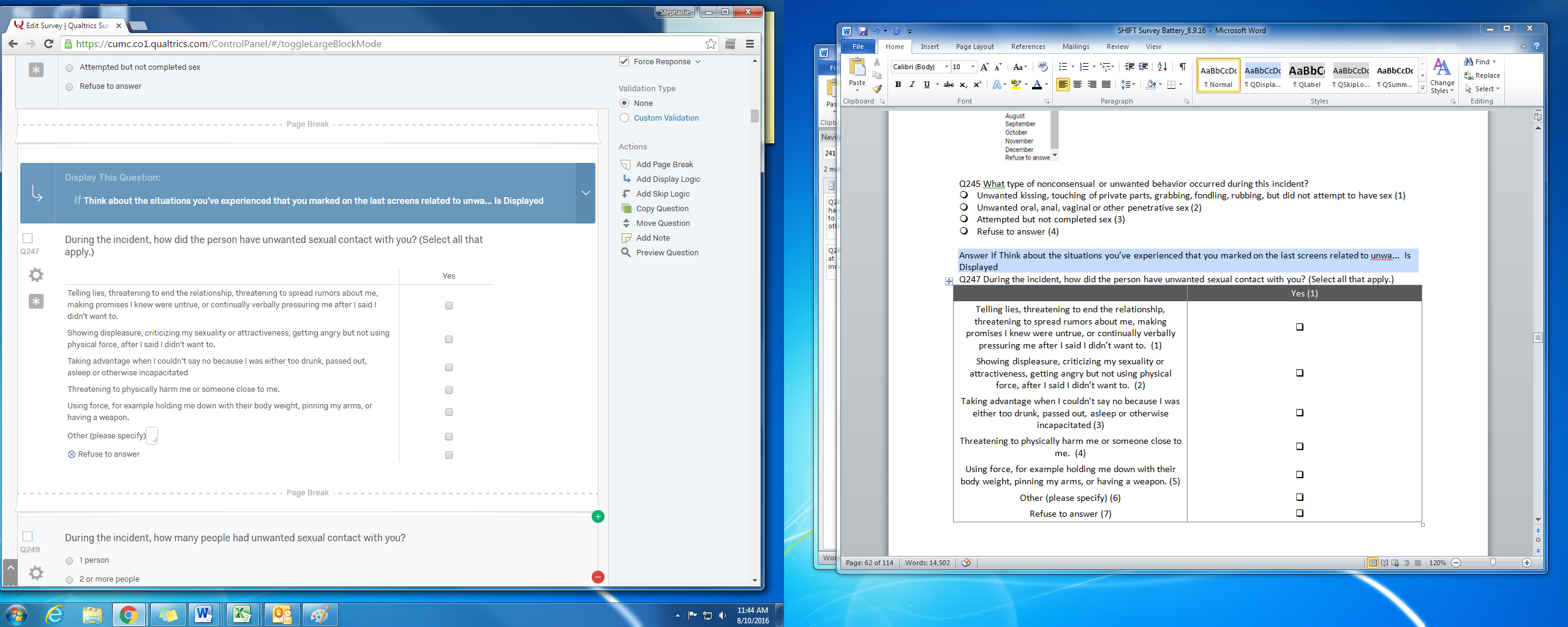

Supplement: S1 Appendix — (DOCX) [file pone.0205951.s002.docx]
