# Supplementary material for: Does sex education before college protect students from sexual assault in college?
Source: PLoS One. 2018 Nov 14;13(11):e0205951. doi: 10.1371/journal.pone.0205951 (PMC6235267; doi:10.1371/journal.pone.0205951)
Supplement: S2 Appendix — (PDF) [file pone.0205951.s003.pdf]

SHIFT
Student In-depth Interview Guide

Background (15 mins)

We are interested in learning about your friendships, social experiences, substance use and experiences with sexual intimacy. But before we dive into that, first I'd like to know a little bit more about you and your background.

1. Tell me a bit about your family and your life before you came to Columbia/Barnard.
   - Who is in family?
   - What are your relationships like with various family members (who close to, who not close to)?
   - What do your parents/other people who raised you do for work?

2. Tell me about your high school
   - Public/private, academically rigorous/not
   - Activities – sports, special skills – what
   - When did you learn about sex, and what kind of sex education have you had?

3. What was your social life like in high school?
   - What kind of person where you in high school? How did you think of yourself, and how would others have described you?
   - Get the story of coming of age, both sexually and with substance use
     - Was there an atmosphere of sexual shame or openness in
       - Family
       - School
       - Peers
     - Things to know about your early sexual experiences (BROADLY understood):
       - How did you feel about them?
       - What kind of health behaviors (protection) did you engage in?
       - How did consent process – expressing and receiving – work?
       - Was there any intertwining of sex and substance use
       - Location of sex - back seat, party, date, prom
       - How interviewee felt about the experience, the person
       - If LGBT: Coming Out story
   - DEPENDING ON HOW TIME IS GOING:
     - Did you ever get in trouble for anything in high school?
     - Did you do anything your parents didn't know about?

Columbia/Barnard story (30 mins):

1. Coming to Columbia/Barnard – why did you come here?
   - Were there any financial aspects to your decision?
   - What were you most excited about before you got here?
   - Looking back, what did you hope to get out of college?

- Probe: socially, academically, professionally

- Did you have an goals, sexually?
- What are your family's hopes/expectations for college?

2. Tell me about your first week here – what do you remember from orientation? [let them answer]
   - Probes about first week:
     - meeting roommates
     - orientation week social experiences
       - Experiences with Sex (and/or expectations)
       - Experiences with Drinking (and/or expectations)
     - Content of orientation. Hidden curriculum about responsible community (sessions that we weren't allowed to observe like where upperclassmen talk to underclassmen). What did they learn about like CONSENT and DRINKING in these sessions?
     - Were there things you did freshmen year that you would never do again? Tell me about them:
       - Get the story: why they did thing, how it felt, consequences

3. College can be so different from high school.
   - Tell me what you found hardest about adjusting to college life.
   - Tell me what was the best, compared to being in high school.

4. It seems to us that people's experiences change a great deal over the time that they are college students, and so I want to get a sense of the overall trajectory of your time here. I'm going to want to go much more in depth about some of these things, but first it would be great if you could give me a rough sketch of where you've lived, your extracurriculars, if you've worked, how you've done academically, how you'd characterize your friend group, your relationship status or hooking up, any mental or physical health challenges you've faced, and what you've done in the summers. At this point, I just want a really rough map, and then we can circle back and go into more specifics…
   - Probes for each year:
     - living circumstances (with whom, where)
     - social life (where, what, with whom)
     - relationship status or hooking up
     - the institutions and activities that they have been a part of academic focus, performance
     - whether and how much they have worked for $
   - Assuming that their current life is different from fall semester freshmen year:
     - why have you made the changes you have made in how you spend your time?

5. Looking back, if you got one do-over, what would it be?
6. What are you most proud of, in your time here?
7. What's been your biggest disappointment, in terms of Columbia/Barnard as institutions – a moment when you really felt like the institution did not work for you?
8. What about a time when you felt that it really did work for you?

<u>Friendships and social life (25 min)</u>

Now I'd like to focus on the present: your activities, friendships, and where and how you socialize now. Tell me about **LAST WEEK**, in terms of how you spent your time.

- Work (how many hours/week, where)
- Studying (where)
    - Why is that a good place to study?
- Eating (meal plan? Food insecurity?)
- Extracurriculars: clubs, teams, organizations, etc.
    - What is satisfying about those things? (why do they spend their time on them?)
    - What is the kind of person who joins that?
- Any part of social life or kind of social event that you actively avoid?
- About how much money would you say you spend each week, between socializing and meals? Where do you get this money
- Socializing:
    - people you hang out with, and what you do with them
        - different groups of friends for different things?
    - Parties and fun times:
        - Spaces, substances, social activities – what have they not said yet about how they socialize?
- Now I'd like to go more in depth about socializing. Tell me the story of a recent weekend (or weeknight) evening that you'd say is typical of how you mostly socialize now with your friends.
    - How do you get ready?
    - Where did you go?
    - How did you afford it?
    - What did you do?
    - Substances (why those, how get them, or why not us them)
    - Was sex, or the hope of sex, part of the evening?
        - Planning on hooking up? Not thinking about it or avoiding it?
        - Preparedness – shaving legs, underwear, condoms, birth control
        - Pre-gaming
- Tell me about a recent time you socialized where it was really fun – what was so fun about it? (doesn't have to involve sex/alcohol)
- A time that was really disappointing or awkward or awful – what made it that way?
- Something that you regretted socially, and why
- Something coming up that you are looking forward to, and why?
- So of the people you hang out with, who do you seek out when you are worried or stressed out about something? Can you give me an example?
    - Are there things that you worry about that you don't really feel like you can share with anyone?
    - Do you feel like you're able to express yourself?

<u>Sex/Intimate relationships and consent (35 MINUTES)</u>

We already talked a bit about sex, but now I want to go into greater detail. For the success of our research, it's extremely important to understand how sex and intimacy work here at Columbia/Barnard. Before I get to those questions, I want to remind you of several of things. First, you are not required to answer any question, and can always ask us to move on to the next question. Second, we take very seriously our obligations regarding confidentiality and that we are exempt from "mandatory reporting." Third, not everyone is pursuing or has experienced sex here on campus, and so we are interested in any kind of intimate relationships you've had that go beyond what you consider friendship. Finally, we have a list of resources we will give you at the

end of this interview should you wish to talk to someone else about your experiences.

1. Are you sexually active?
2. Tell me about your sex/intimate life.
    - What are you looking for?
        - Intimacy, pleasure, do you have concerns at all about reputation
        - Describe your best sexual/intimate experience in your time at Columbia/Barnard
            - What happened?
            - Circumstance around it?
            - Did you use protection? (if sex)
            - Who did you tell about it?
            - Where, and how negotiate in terms of roommate?
            - How meet partner – social media, student activity, party, bar, etc.?
    - Do you "hook up" or have you before?
        - If no: why not?
        - How does it happen? (social media) – which one, what look for?
        - What does it satisfy?
        - Are there things that you imagine getting from sex that it does not satisfy?
        - Who is an ideal hookup partner?
        - Bad hookup story
        - Good hookup story

If relationship:
What is the story of the relationship? How meet, how court, how end, etc.

From totally casual to super committed, what are the range of kinds of sexual experiences and relationships that you have had as a student?

Probe on those.

- Partnership ideals
- Seeking vs. being sought
- The role of sex in that relationship – or, thinking about it a different way, what would it be without sex?
- How does sex fit into your life overall here?
- What is the relationship between alcohol and your sex life?

REGRET
Have you ever had a sexual [INTIMATE] experience that you regretted?

- When did you first begin to regret it?
- Did you do anything about it or talk to anyone about it?
- Did you ever have later conversations with a partner that made you reconsider the character of a sexual encounter? Where perhaps they regretted it?
- What was the result?
- Can you tell me about the worst sexual/intimate relationship you've had?

How have you conveyed to your partners about things you like? How have

you convey to your partners about things you don't like?

What are the things you worry about when you have sex? LET THEM ANSWER. If they don't have an answer, or once they answer, give them some examples. Such as, do you ever worry about:

- Body image
- Not "good" at sex
- How people will think of you after sex
- STIs
- Not knowing how to convey consent?
- Does alcohol ever help you manage some of these concerns?

CONSENT

For the sexual or intimate experiences you told me about, I want to know how consent worked. [if there are many, ask about one relationship and one hookup]

- In X sexual encounter, how did you convey consent to your partner(s)?
- How did you continue to convey consent as physical contact continued?
- How did your partner(s) convey consent to you? (GENDER DYNAMICS?)
- In sexual or intimate relationships where you did not receive affirmative consent (verbal consent for each progressive act), how did you know the contact was consensual?
- In sexual or intimate relationships where you did not grant affirmative consent, how do you think your partner knew the contact was consensual?
- Have you ever had a sexual or intimate experience that ended before one person wanted it to because one of you did not grant consent or withdrew it?
    - How did that work?
    - When people change their mind about what they want to do after something has already begun, what do they do?

- Have you ever reflected back on a sexual or intimate encounter and felt that you might not have fully granted consent, or been concerned that your partner did not fully grant consent? Tell me about that.
    - What made you feel uncomfortable afterwards?
    - What, if anything, did you do about it?
- Would you describe this "uncomfortable nonconsensual" sexual experience as sexual assault, either as victim or perpetrator?
    i. If no: why not? Then follow with probes below
    ii. If yes:
    - Can you describe the emotional experience you went through (continue to experience)?
    - Who did you tell, and who didn't you tell about it?
        - Tell me about how that went
    - What did you need to help you make sense of this experience?
    - What did you get, compared to what you needed?
    - [IF PERSON WAS PERPETRATOR]:
        - How do you think it happened?
        - How do you feel or think about the experience or yourself since?
    - What do you know about the process of reporting sexual violence here at Columbia? How did you learn about this?

How about your friends – do you know people who have experienced sexual contact against

their wishes or while they were too intoxicated to give consent?

- Can you share any stories about the experience of reporting sexual violence?
- What happened?

Institutional Experiences (10 minutes)

Finally I'd like to end by talking about your experiences with Columbia/Barnard as an institution. During your time here as a student you have probably interacted with a range of staff people and administrators, and of course with many faculty.

- Tell me about some of the positive relationships or interactions that you've had with people who work for Columbia/Barnard.

- Tell me about some of the disappointing relationships or interactions that you've had with people who work for Columbia or Barnard.

- If you needed advice or help, who is the first person you'd talk to?
  - Is there anything you wouldn't talk to this person about?

- If you needed advice from someone older, who isn't a peer, who would you go to?
  - Who would you never go to?

If you needed help is there someone you'd feel comfortable talking to who is: (get story as to why/why not):

- Professors/TA
- Health Services
- Office of Disability Services
- Public Safety
- Office of G-B Misconduct
- Title IX Coordinator
- SVR
- Athletic Department
- Any other institutional actor

Final statement (5 minutes)

Thank you so much for your time. This has been extremely helpful. My main goals for this interview were to understand:

1. What your life here at Columbia.
2. What your sexual and intimate relationships are like here at Columbia, how you've developed, sexually, and how consent works within those relationships.
3. How institutional arrangements at Columbia might influence your life as a student

Is there anything I should have asked you in relationship to these three questions or anything else that I didn't cover?
